# Microbial community changes along the active seepage site of one cold seep in the Red Sea

**DOI:** 10.3389/fmicb.2015.00739

**Published:** 2015-07-21

**Authors:** Huiluo Cao, Weipeng Zhang, Yong Wang, Pei-Yuan Qian

**Affiliations:** ^1^Division of Life Sciences, The Hong Kong University of Science and TechnologyClear Water Bay, Hong Kong; ^2^Sanya Institute of Deep Sea Science and Engineering, Chinese Academy of SciencesSanya, China

**Keywords:** cold seep, Red Sea, 16S rRNA gene, pyrosequencing, ammonia oxidizing archaea

## Abstract

The active seepage of the marine cold seeps could be a critical process for the exchange of energy between the submerged geosphere and the sea floor environment through organic-rich fluids, potentially even affecting surrounding microbial habitats. However, few studies have investigated the associated microbial community changes. In the present study, 16S rRNA genes were pyrosequenced to decipher changes in the microbial communities from the Thuwal seepage point in the Red Sea to nearby marine sediments in the brine pool, normal marine sediments and water, and benthic microbial mats. An unexpected number of reads from unclassified groups were detected in these habitats; however, the ecological functions of these groups remain unresolved. Furthermore, ammonia-oxidizing archaeal community structures were investigated using the ammonia monooxygenase subunit A (*amoA*) gene. Analysis of *amoA* showed that planktonic marine habitats, including seeps and marine water, hosted archaeal ammonia oxidizers that differed from those in microbial mats and marine sediments, suggesting modifications of the ammonia oxidizing archaeal (AOA) communities along the environmental gradient from active seepage sites to peripheral areas. Changes in the microbial community structure of AOA in different habitats (water vs. sediment) potentially correlated with changes in salinity and oxygen concentrations. Overall, the present results revealed for the first time unanticipated novel microbial groups and changes in the ammonia-oxidizing archaea in response to environmental gradients near the active seepages of a cold seep.

## Introduction

Cold seeps mainly occur in geologically active and passive continental margins, and they transport dissolved and gaseous phase compounds to the ocean to sustain significant chemosynthetic biomass by providing bioactive reductants, sulfides, methane and hydrogen (Levin, 2005; Suess, 2010). Many early studies have focused mainly on carbon and sulfur cycling in these specialized ecosystems, with a specific focus on the anaerobic oxidation of methane (AOM) coupled to sulfate reduction (SR) in microbes in hypersaline cold seep sediments (Maignien et al., 2013). In particular, AOM in a cool seep environment has been extensively studied using metagenomic and metatranscriptomic methods (Stokke et al., 2012), while changes in the microbial composition along the environmental gradient near the seepage sites have only been marginally assessed.

In cold seep ecosystems, microbes must rapidly transform carbon, sulfur and nitrogen compounds. Nitrogen fixation plays important roles in cold seeps (Pernthaler et al., 2008; Dang et al., 2009; Dekas et al., 2009; Miyazaki et al., 2009), and high rates of nitrogen removal due to denitrification in cold seep sediments have been proposed (Bowles and Joye, 2011). The diversity and abundance of anaerobic ammonium oxidizing (anammox) bacteria in cold seep hydrocarbon-rich fluids have been reported (Russ et al., 2013; Shao et al., 2014). A reduced diversity and abundance of the ammonia oxidizing archaea (AOA) thaumarchaea were found in cold seep sediments in the Okhotsk Sea (Dang et al., 2010) and northeastern Japan Sea (Nakagawa et al., 2007). All of these studies have shed some light on the possible contributions of various types of microorganisms to nitrogenous nutrient recycling; however, the ecological functions of the thaumarchaea in cold seeps remain largely unresolved.

The oxidation of ammonia is the first and rate-limiting step in the process of nitrification (Kowalchuk and Stephen, 2001) performed by bacterial and archaeal groups. Ammonia monooxygenase (amo) catalyzes the oxidation of ammonia and consists of several subunits, among which the *amoA* gene encoding subunit A has been widely used as a reliable genetic marker to explore the diversity and abundance of AOA (AOA) in diverse ecosystems (Junier et al., 2010; Cao et al., 2011b). Various environmental parameters, such as pH, depth, nutrients, and dissolved oxygen, have been identified as potential factors determining the dominant ammonia oxidizer phylotypes and their diversity in ecosystems (Erguder et al., 2009; Cao et al., 2013). However, the ecological role of the AOA in cold seeps remains unexplored.

The Thuwal Seeps is a cold brine seep system located at a depth of ∼850 m and was first discovered on May 7, 2010 by a remotely operated vehicle (ROV) on the Saudi continental margin of the central Red Sea during a survey built into the framework of “KAUST Red Sea Expedition Spring 2010” (Batang et al., 2012). The seep is located at the base of a steep rocky wall that is closer to the shore (20 km) than to the axial trough (120 km). In fact, active brine ventings have been observed at two seep sites, which are named Thuwal Seeps I and II (I is located at 22° 17.3′ N–38° 53.8′ E; II is located at 22° 16.9′ N–38° 53.9′ E). A shallow brine pool was formed by fluids from seeps with a low temperature (21.7°C) and salinity (74‰) compared with the other brine pools in the Red Sea (Batang et al., 2012). Although the hypersaline brine pools at the Thuwal Seeps are harsh to organisms, high biomass production was observed (Batang et al., 2012). Brine waters likely originate from evaporitic deposits of submarine geological formations that flow from the faulting system at the base of the rocky scarp where the Thuwal brine pool formed. Extended chemosynthetic bacterial mats and dense aggregations of live and dead organisms have been observed (spatangoid urchins, anemones, serpulid tubeworms, sponges, clams, fishes, crabs and shrimps) (Batang et al., 2012). Thus, the Thawal seeps provides a good opportunity to understand the responses of microbes that reside near the seepage sites of cold seeps.

To explore potential changes in the microbial communities from the seepage site to nearby areas in this cold seep ecosystem, habitats including marine sediments, marine water, seep water, and microbial mats were sampled for 16S rRNA gene analysis. Because high concentrations of inorganic nitrogen species were detected in the environment, the AOA community structures were also examined based on the *amoA* gene. The results showed that the habitat type dictated the community structure, while the environmental gradient shaped the changes in the AOA community from the active seepage site to peripheral areas.

## Materials and Methods

### Sampling and Environmental Parameter Measurements

**FIGURE 1:**
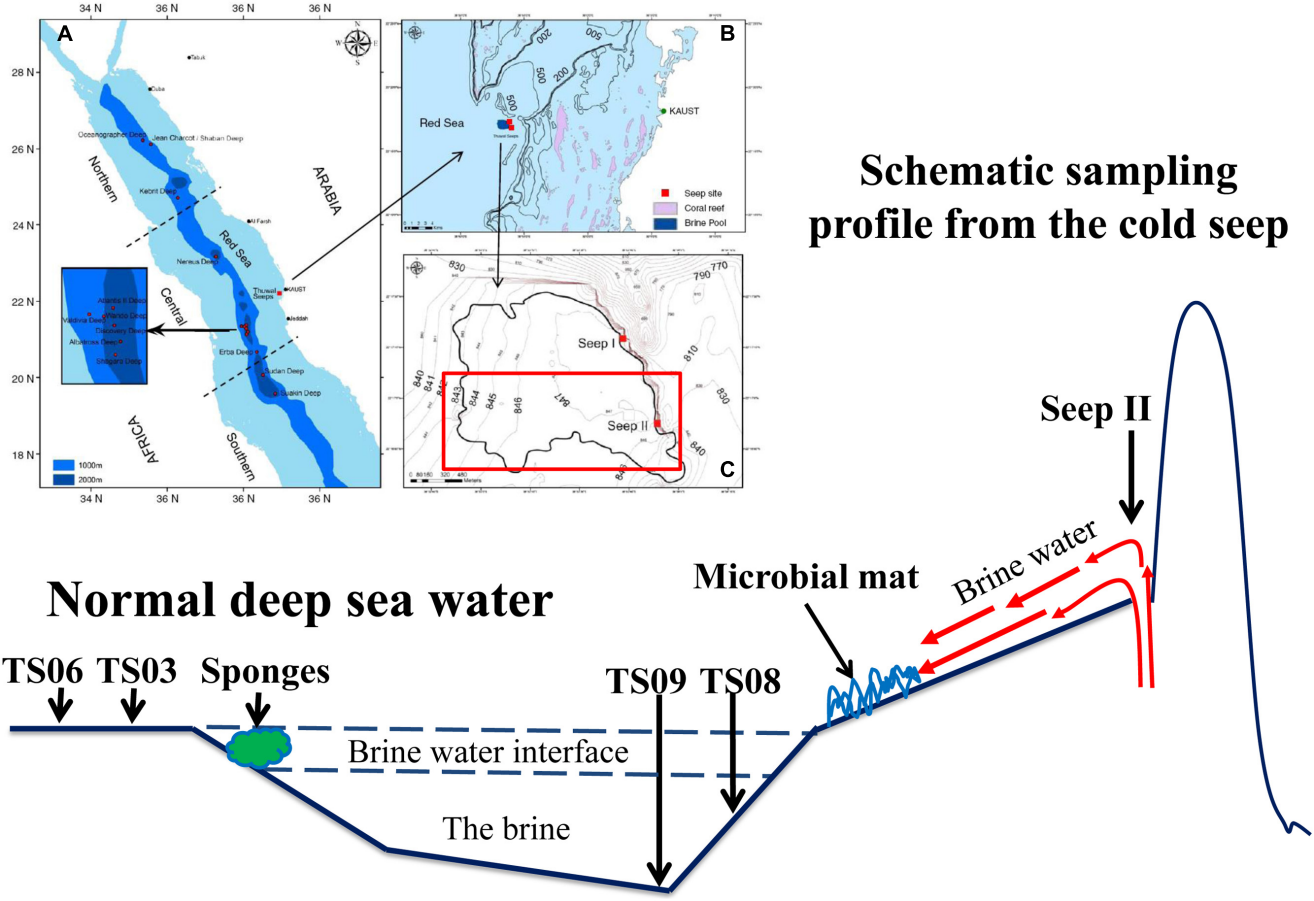
**Diagram of the sampling sites in the Red Sea modified from Batang et al. (2012). (A)** Locations of brine pools in the Red Sea axial zone (blue shade); **(B)** Location of Thuwal Seeps (dark blue patch) near coral reefs (pink patches) along the Saudi coast; **(C)** Outline of Thuwal Seeps brine pool (thick line) superimposed on depth contours (Batang et al., 2012).

Field sampling was conducted in November 2011 in the Thuwal cold Seep II (22^o^16’N–38^o^53’E) via the ROV *Max Rover* developed by Deep Sea Systems International (DSSI), USA, during the KAUST Red Sea exploration cruise (**Figure 1**). The venting site of the seepage was only about 1.5 m wide and 1.0 m deep, while the brine pool was very shallow with a depth of approximately 1.0 m in most places. Four types of habitats around the brine pool approximating the seeping vents were sampled. The samples from the seep vent (Seep4), normal marine water (TS06W, normal marine water overlaying the pool), sediments (TS03S and TS06S from outside the brine pool; TS08S from inside the brine pool), and a microbial mat on the bank of the pool (top of the microbial mat) have been described in a previous study (Wang et al., 2014b). Two replicate each were sampled from the seep and marine water for 16S rRNA gene analysis.

The *in situ* physicochemical parameters (temperature, salinity, and concentration of dissolved oxygen) were measured as described previously (Lee et al., 2014; Wang et al., 2014b). The concentrations of dissolved organic carbon (DOC) and total nitrogen (TN) in the water sample and pore-water from the sediment were measured using the combustion method (Trichet et al., 2001), while the concentrations of ammonium and nitrate plus nitrite were determined separately using a TNM-I analyzer (Simadzu, Kyoto, Japan). Detailed descriptions of the sampling sites and their physicochemical characteristics are provided in **Figure 1** and **Table 1**.

**Table 1:** Physicochemical parameters of the profiles from the cold seep to the far sediments in the Red Sea (some samples and parameters were adopted from Wang et al., 2014b).

Sample ID	Descriptions	Coordinate	TOC (mg/L)	TN (mg/L)	O_2_ (%)	Salinity (‰)	NH_4_^+^ (μM)	NO_2_^-^+NO_3_^-^(μM)
Top	Top of the microbial mat	22°16.999N- 38°53.893E	35.95	7.87	25	43	–	–
TS06W	Deep sea water	22°17.100N- 38°53.725E	19.22	2.60	25	43	22.94	1.45
Seep4	Seep water	22°17.042N- 38°53.897E	26.12	27.22	<0.2	125	386.66	3.24
TS03S	Deep sea sediment	22°17.100N- 38°53.075E	76.38	12.49	24.4	43	246.40	2.18
TS06S	Deep sea sediment	22°17.100N- 38°52.90E	71.04	5.48	25	43	65.25	4.83
TS08S	Brine pool sediment	22°17.210N- 38°53.736E	60.88	17.13	~0.5	96	456.46	2.90
TS09S	Brine pool sediment	22°17.313N- 38°53.645E	58.03	16.87	0.2	96	705.15	2.73

### Molecular Experiments

Genomic DNA was extracted from all of the sediment samples (collected in triplicate) using the PowerMax Soil DNA Isolation Kit (Epicentre Biotechnologies, Madison, WI, USA) according to the manufacturer’s instructions. For microbial mat and water filters, the modified SDS-based method described by Lee et al. (2009) was employed. The DNA quality and quantity were checked using a PicoGreen dsDNA quantitation kit (Life Technologies, Carlsbad, CA, USA) and gel electrophoresis.

The *amoA* gene was cloned into a plasmid, and 16S rRNA gene sequencing was conducted by pyrosequencing. PCR amplifications using the isolated genomic DNA as template were performed in 20-μl reactions consisting of 1.25 U of Taq DNA polymerase (New England Biolabs, England), 2 μl of 10× PCR buffer (15 mM Mg^2+^), 1 μl of deoxynucleoside triphosphates (dNTPs) (2.5 mM), 2 μl (∼30 ng) of DNA template, and 0.5 μl of each primer (10 μM) (Arch-amoAF: 5′-STAATGGTCTGGCTTAGACG-3′ and Arch-amoAR: 5′-GCGGCCATCCATCTGTATGT-3′) (Francis et al., 2005). The amplicons resulting from PCR performed in triplicate for each sample were pooled and used to construct clone libraries. The amplified 635-bp bands were excised from 1.0% (wt/vol) agarose gels and gel-purified using the Universal DNA Purification Kit (Tiangen Biotech, China). The purified PCR products were cloned into the pCR2.1-TOPO vector using the TOPO TA cloning kit for sequencing with One Shot TOP10 competent cells according to the manufacturer’s instructions (Invitrogen, USA). Each clone was randomly selected and screened by PCR with primers M13F and M13R to select positive clones. The positive clones were sequenced with the vector-specific primers M13F and M13R using the Applied Biosystems 3730xl DNA analyzer (Applied Biosystems, Foster City, CA, USA).

Regarding the 16S rRNA gene pyrosequencing, the hyper variable regions V4 to V8 of the bacterial and archaeal 16S rRNA gene were amplified for each sample using the universal forward primer U515F (5′- GTGYCAGCMGCCGCGGTAA -3′) and the reverse primer R1492 (5′- GACGGGCGGTGTGTRCAA -3′) (Wang et al., 2014a; Zhang et al., 2014) with unique 6-nucleotide (nt) barcodes. Five units of *Pfu* Turbo DNA polymerase (Stratagene, La Jolla, CA, USA), 1x *Pfu* reaction buffer, 0.2 mM dNTPs (TaKaRa, Dalian, China), 0.1 μM each barcoded primer, and 20 ng of genomic DNA template were mixed in a 100-μl PCR volume. The PCR procedure included an initial denaturation at 94°C for 5 min, 26 cycles at 94°C for 30 s, 53°C for 30 s, and 72°C for 45 s, and final extension at 72°C for 6 min using a Bio-Rad thermal cycler (MJ Research Inc., Bio-Rad). The PCR products were purified using a TaKaRa Agarose Gel DNA Purification Kit (TaKaRa, Dalian, China) and quantified with a NanoDrop device. Two hundred nanograms of each purified 16S amplicon were mixed and then subjected to pyrosequencing using the Roche 454 FLX Titanium platform at the Chinese National Human Genome Centre in Shanghai, China.

### Sequencing Analysis for 16S rRNA Gene Pyrosequencing Data

The pyrosequencing data were deposited in the NCBI Sequence Read Archive (SRA) database. The downstream bioinformatics analysis was performed using QIIME 1.7.4 (Caporaso et al., 2010b). The criteria used for quality control of all data have been described in a previous study (Lee et al., 2012).

Reads were assigned to their respective samples according to their barcodes and then subjected to a second round of quality control using Denoiser (Reeder and Knight, 2010). Qualified reads were clustered using uclust (Edgar, 2010) and then assigned to operational taxonomic units (OTUs) at a similarity of 97%. Representatives of the most abundant reads were selected from each OTU for subsequent analysis. Representative OTUs were aligned *de novo* using MUSCLE (Edgar, 2004), and a phylogenetic tree was produced using FastTree (Price et al., 2009). Representative OTUs were also aligned using PyNAST (Caporaso et al., 2010a) with the Silva108 database as a reference. Successfully aligned reads were submitted to ChimeraSlayer (Haas et al., 2011) to identify and discard chimeric reads. Species diversity, richness, and rarefaction curves were computed at a similarity of 97% as part of QIIME’s alpha diversity pipeline. Beta diversity analysis was conducted after rarefying the samples in the smallest library using QIIME. A step size of 100 was used with 100 repetitions at each step. Taxonomic assignment was conducted using the Ribosomal Database Project (RDP) classifier version 2.2 (Wang et al., 2007) against Silva108 (Pruesse et al., 2007) with a bootstrap confidence level of 50%. The number of reads assigned to the different genera were converted into percentages.

### Diversity Indice Calculations for the *amoA* Gene

The DNA sequences of the *amoA* genes were transformed into MEGA 6.0 software (Tamura et al., 2013) and aligned using CLUSTALW in MEGA. OTUs were identified at a similarity level of 0.97 using the MOTHUR program (Schloss et al., 2009). Based on the OTU assignments, the nonparametric abundance estimators, Chao1 and ACE, and the diversity estimates (ACE) were calculated using *summary.single* in MOTHUR. Rarefaction curves were calculated for all samples at a distance cutoff of 0.03 using *rarefaction.single* (Schloss et al., 2009).

### Phylogenetic Analysis of the *amoA* Gene

The selected OTUs from all the samples were imported into MEGA and translated into amino acids. Reference sequences from GenBank were downloaded to construct a comprehensive database and then clustered at an amino acid similarity level of 0.97. After merging with the reference sequences, including those from enrichments, the initial phylogenetic tree was constructed based on the neighbor-joining (NJ) algorithm with 1,000 bootstrap replicates using MEGA software (Tamura et al., 2013). The NJ phylogenetic tree was employed initially to construct the maximum likelihood (ML) tree in MEGA.

### Microbial Community Analyses for the *amoA* Gene

To compare the phylogenetic diversity between different habitats, a genetic distance matrix of the sequences from each habitat was developed. Principal coordinate analysis (PCoA) and jackknife environment cluster analysis were conducted using the online software Fast UniFrac (Hamady et al., 2010), which utilizes the genetic distance matrix based on the gene sequence data. In addition, hierarchical clustering analysis (UPGMA algorithm with jackknife supporting values) was used. The environmental cluster tree was projected in MEGA 6.0 (Tamura et al., 2013).

Non-metric multidimensional scaling (NMDS) was conducted in MOTHUR based on a genetic cutoff distance of 0.03 for *amoA* to determine the similarity between samples. The command was modeled based on the function using the majorization algorithm (Borg and Groenen, 1997).

One-way ANOSIM methods with 999 permutations were performed in MOTHUR to test for the significance of differences in community composition between the clone libraries. Simultaneously, LIBSHUFF statistical comparisons were conducted using LIBSHUFF in MOTHUR.

### Nucleotide Sequence Accession Numbers

All of the archaeal *amoA* gene sequences retrieved in the present study have been deposited in the GenBank database at NCBI under accession numbers KM109433–KM109966, and the 16S rRNA gene pyrosequencing data have been submitted to the NCBI SRA database under accession number SRX501833.

## Results

### Sampling Site Descriptions and Physiochemical Parameters

The features of our sampling sites have been documented in a previous study (Wang et al., 2014b), and the sampling map is also shown in **Figure 1**. Higher concentrations of inorganic nitrogen compounds have been observed in the seep and site TS08S compared with sites TS03S and TS06 (**Table 1**). High concentrations of hydrogen sulfide (>200 μmol/L) were also recorded in the nearly anoxic brine pool (Wang et al., 2014b).

### 16S rRNA Gene Diversity and Composition

A total of 47,353 reads (∼4304 reads per sample) were passed through the quality check. The number of OTUs and estimated species richness at the 3% dissimilarity level are listed (Supplementary Figure S1). The highest number of OTUs was found on top of the microbial mat followed by the marine sediments (Supplementary Figure S1).

At the 50% confidence threshold, qualified reads could be assigned to eighteen phyla based on the analyses using the QIIME pipeline (Supplementary Figure S2). The proportions of these phyla varied among different habitats. For example, 12.70% of the Proteobacteria was observed in TS06W-1. In contrast, Proteobacteria demonstrated a low abundance in marine sediments but were exceptionally enriched in the microbial mat. Marine sediments contained a higher abundance of Euryarchaeota than the seeps and marine water (<0.25%). The Thaumarchaea was an over-represented phylum in seeps and marine water compared with marine sediments and the microbial mat (Supplementary Figure S2). Nitrospirae were highly abundant in two marine sediment samples, TS08S and TS09S, whereas a high abundance of Deferribacteres was detected in the seeps and marine water (Supplementary Figure S2).

**FIGURE 2:**
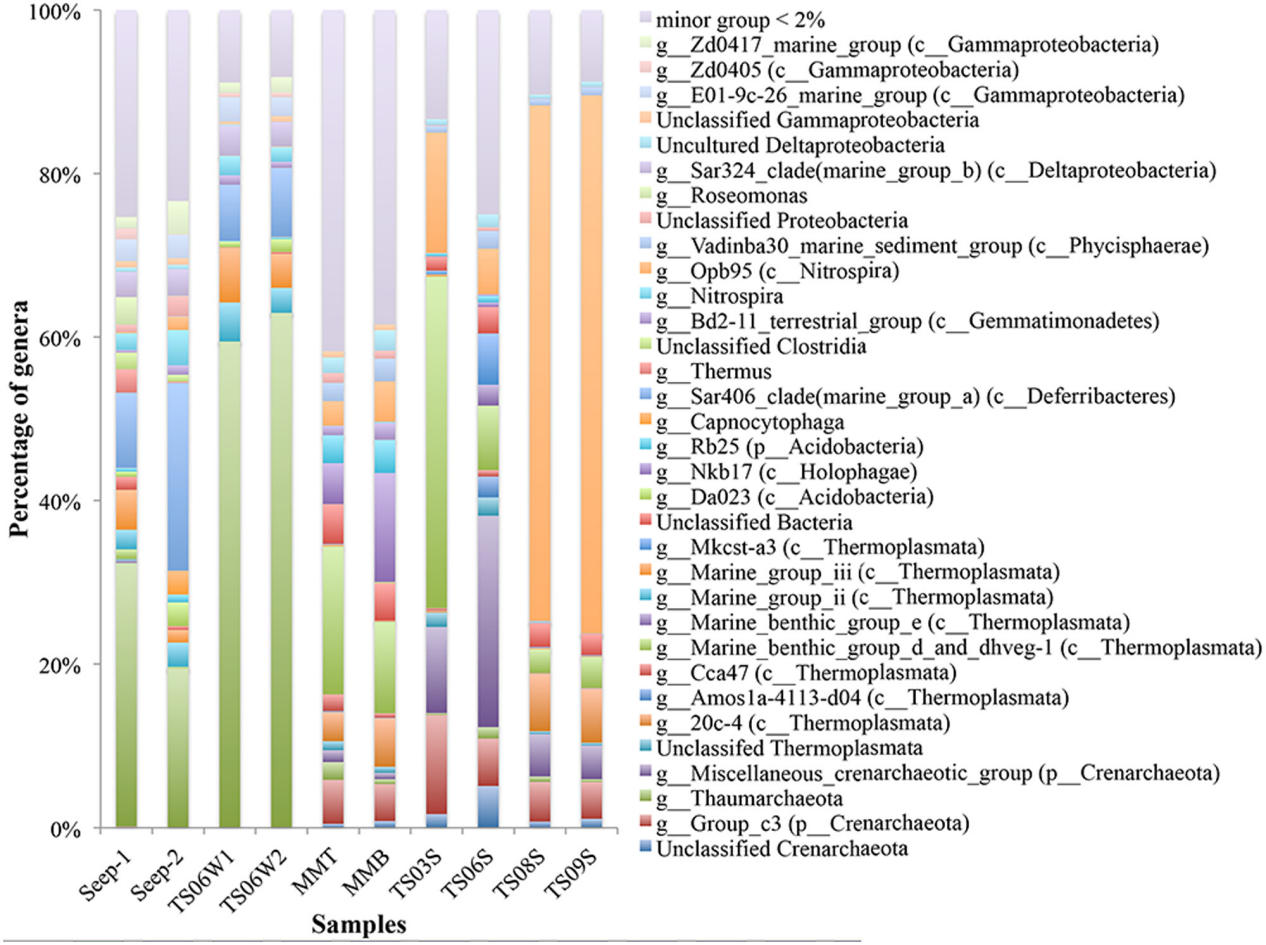
**Taxonomic classification at the genus level of microbial reads retrieved from different habitats in the Red Sea based on 16S rRNA gene pyrosequencing data presented as the relative abundance (MMT and MMB denote the top and bottom of the microbial mat, respectively)**.

Substantial variations in the microbial communities associated with different habitats were detected down to the genus level. For example, the microbial communities from marine water and seeps were dominated by unclassified Thaumarchaea, ranging from 19.27% in Seep4-2 to 62.50% in TS06W-2 (**Figure 2**). In contrast, marine sediment TS09S and the top of the microbial mat contained proportions of unclassified Thaumarchaeota of 0.34% and 2.17%, respectively (**Figure 2**). In cold seeps and marine water, Thermoplasmata, Sar406 clade (Deferribacteres), *Nitrospira*, Sar324 clade and the E01-9c-26 marine group also exhibited a high abundance. In particular, the abundance of Sar406 was rather high in the seeps (**Figure 2**). Group_c3 in the Crenarchaeota, a miscellaneous crenarchaeotic group, marine benthic group d (MBGD) and dhveg-1 (currently designated as Thermoplasmata) were found in microbial mats and marine sediments. Opb95 (*Nitrospira*) was the dominant species group in two of the sediment samples from the brine pool (TS08S and TS09S) (**Figure 2**). The miscellaneous crenarchaeotic group was dominant in normal marine sediment samples (TS03S and TS06S) (**Figure 2**). In addition, TS03S was dominated by MBGD and the dhveg-1 (Thermoplasmata) group. At the genus level, another two groups, Nkb17 (Holophagae) and Rb25 (Acidobacteria), were also observed with a higher abundance in microbial mats than in the other samples.

### *amoA* Gene Diversity

**FIGURE 3:**
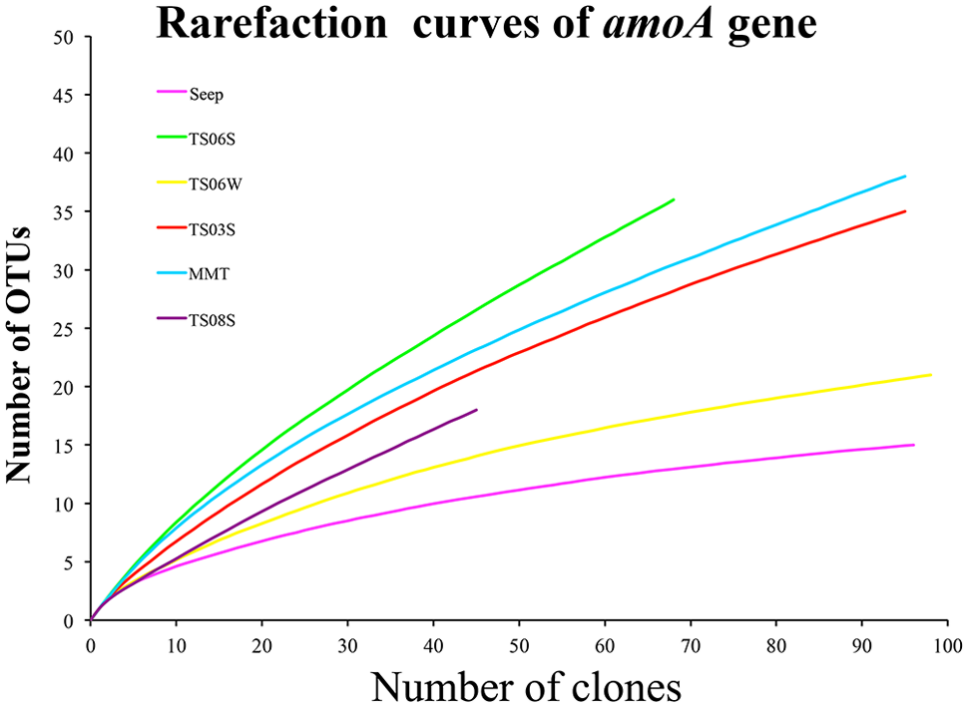
**Rarefaction cures for *amoA* genes sequences from each sample based on a cutoff of 0.03 generated by MOTHUR**.

Rarefaction curves (**Figure 3**) and diversity indices (**Table 2**) were determined for each clone library. The results revealed that the AOA diversity was far from exhaustively sampled, in particular for the microbial mat sample (**Figure 3**). The highest diversity indices were found on the top of the microbial mat and in the marine sediments, which indicated that the sediment may represent the largest reservoir of AOA diversity (**Table 2**). In contrast, the diversity indices were low in water samples from seeps, brine, and normal bottom seawater (**Table 2**).

**Table 2:** Diversity indices for the *amoA* genes in all samples from the Red Sea.

Sample	No. of Clone	operational taxonomic units (OTUs)	Chao	Shannon	Simpson	Coverage (%)
Seep4	96	15	18.75	1.79	0.25	93.8
TS06W	98	21	32.25	2.03	0.26	89.8
TS03S	95	35	63.88	2.73	0.15	76.8
TS06S	68	36	153.00	3.23	0.04	60.3
Top	95	38	88.00	3.13	0.06	82.1
TS08S	45	18	70.50	1.91	0.31	66.7

### Phylogenetic Tree Based on the *amoA* Gene

**FIGURE 4:**
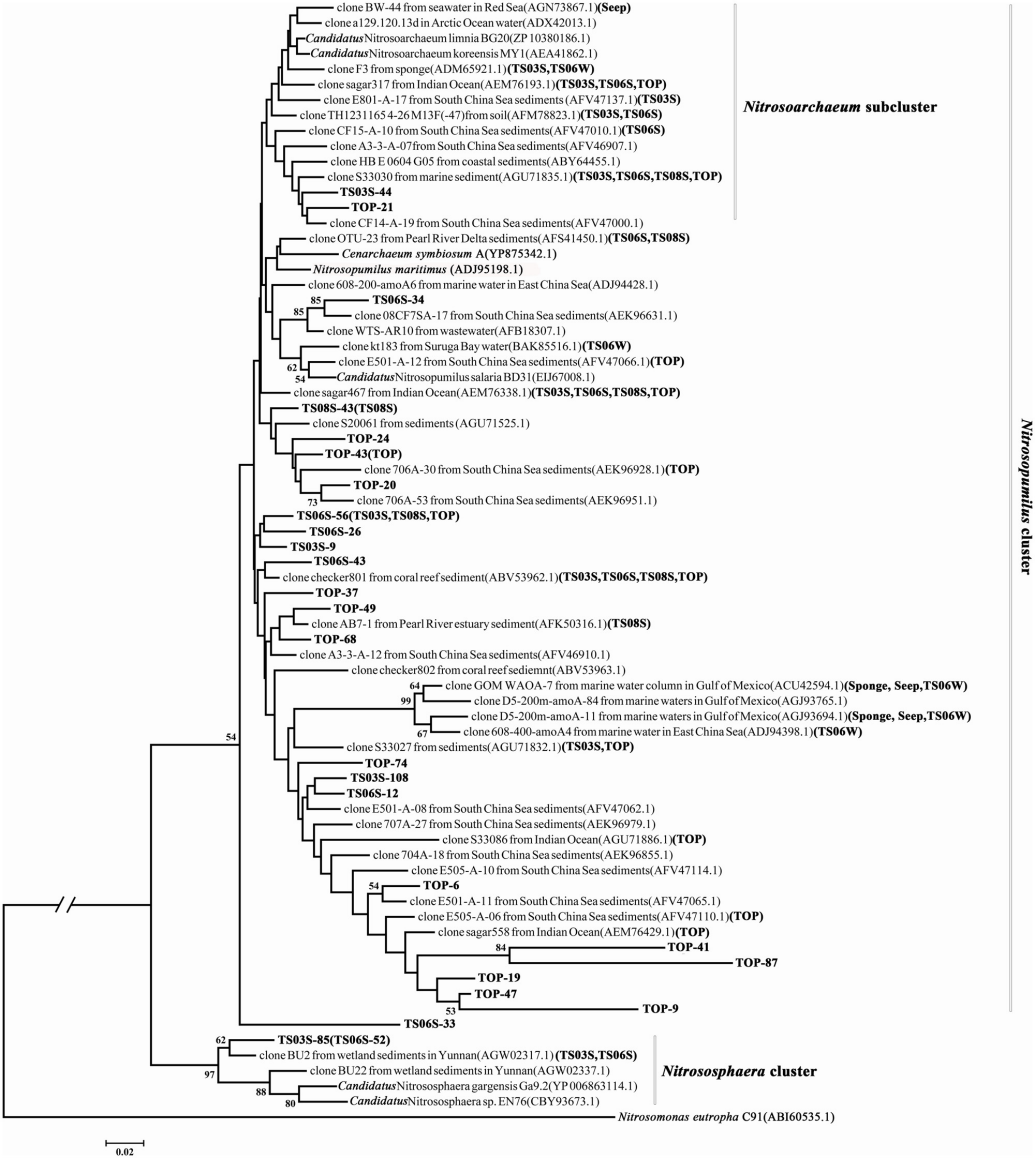
**hylogenetic tree reconstructed from the deduced AmoA protein sequences using the maximum likelihood (ML) criterion.** AmoA sequences from the present study are shown in bold, and sample names of representative sequences within the same operational taxonomic units (OTUs) are bracketed.

The phylogenetic analysis of the *amoA* gene revealed three major monophyletic clusters (i.e., *Nitrosopumilus, Nitrosotalea, Nitrosocaldus*) and a non-monophyletic cluster (*Nitrososphaera*) that comprised mostly genes from the soil and sediment environments (Pester et al., 2012; Cao et al., 2013). In addition, one subcluster, *Nitrosoarchaeum* within the *Nitrosopumilus* cluster, was characterized by organisms that survive in low salinity habitats. In the present study, only two sequences (TS03S-85 and TS06-52) were classified in the *Nitrososphaera* cluster, while both the *Nitrosotalea* and *Nitrosocaldus* clusters were absent (**Figure 4**). In contrast, clusters of *Nitrosopumilus* and *Nitrosoarchaeum* were dominant in this environment (**Figure 4**). Although the statistical supports for most of the nodes were weak, based on two criteria, ML and NJ, the topologies were remarkably consistent with one another. This result supported the stability of the topology, and subsequently, only ML phylogeny was presented (**Figure 4**).

Most of the *amoA* gene sequences determined in the present study was distributed into the *Nitrosopumilus* cluster, with the highest contribution from marine sediments and the top of the microbial mat. Within the *Nitrosopumilus* cluster, most of the subclusters had low support values (**Figure 4**). We downloaded all of the archaeal *amoA* gene sequences from GenBank to create one database (updated to November, 2013) and would assign the gene sequences from the present study to the closest relatives. However, the closest relatives to most of the sequences from the present study affiliated with the *Nitrosopumilus* cluster could not be identified (**Figure 4**). In particular, the sequences from the top of the microbial mat did not cluster with the sequences from GenBank (**Figure 4**). This over-dispersion of sequences from marine sediments and the microbial mat in the *Nitrosopumilus* cluster might be explained by the influence of the chemocline in the sampling area. Sequences from the microbial mat exhibited a higher diversity than those from other habitats and were dispersed throughout the whole phylogenetic tree, although a low abundance of Thaumarchaea was observed in these two samples based on 16S rRNA gene analysis (**Figure 2**). In addition, one lineage with a long branch included a partial sequence of TS06W from normal marine water, and most sequences from the seep clustered with those from marine water in the Gulf of Mexico (Tolar et al., 2013) and East China Sea (Hu et al., 2011) (**Figure 4**).

The subcluster (designated as the *Nitrosoarchaeum* subcluster) in the *Nitrosopumilus* cluster has been previously proposed to reside in a Low Salinity Environment Cluster that includes *Candidatus Nitrosoarchaeum limnia* (Mosier et al., 2012) and *Candidatus Nitrosoarchaeum koreensis* (Kim et al., 2011), which were isolated from low salinity environments (Cao et al., 2013). Surprisingly, in the present study, the sequences from all of the high salinity samples were embedded in this subcluster (**Figure 4**).

### Phylogenetic Ecology of the *amoA* Gene

**FIGURE 5:**
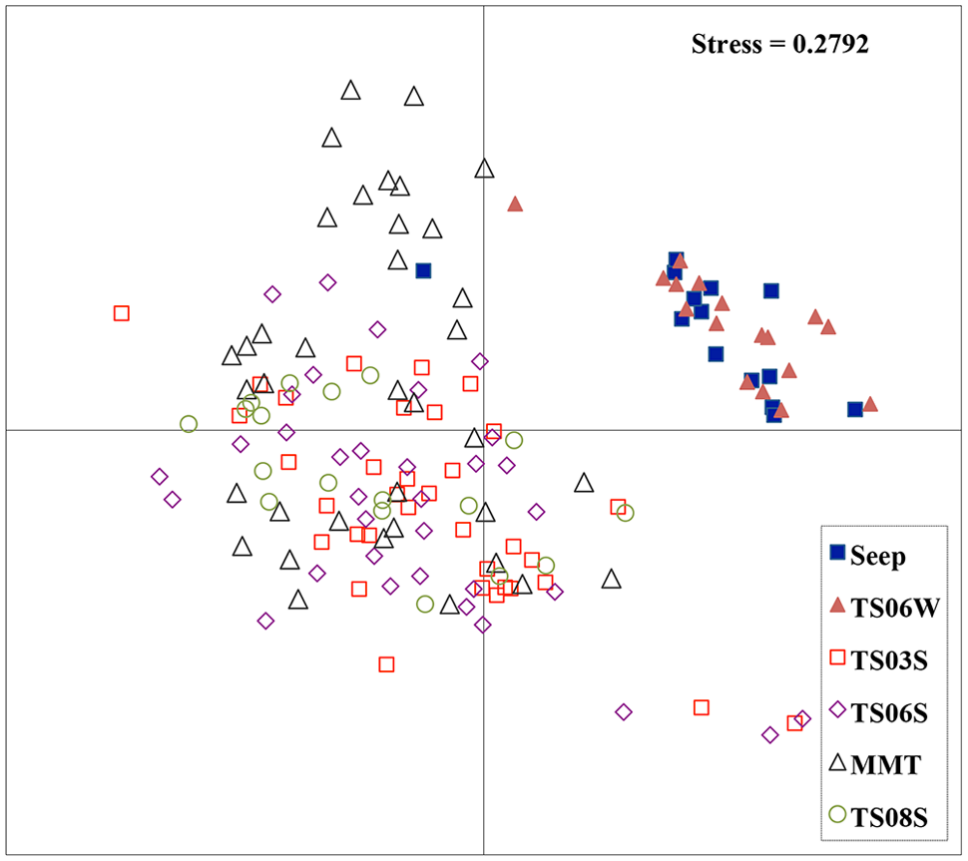
**Non-metric multidimensional scaling (NMDS) function analysis of all *amoA* genes in the present study.** Each point represents one OTU of the *amoA* gene.

**FIGURE 6:**
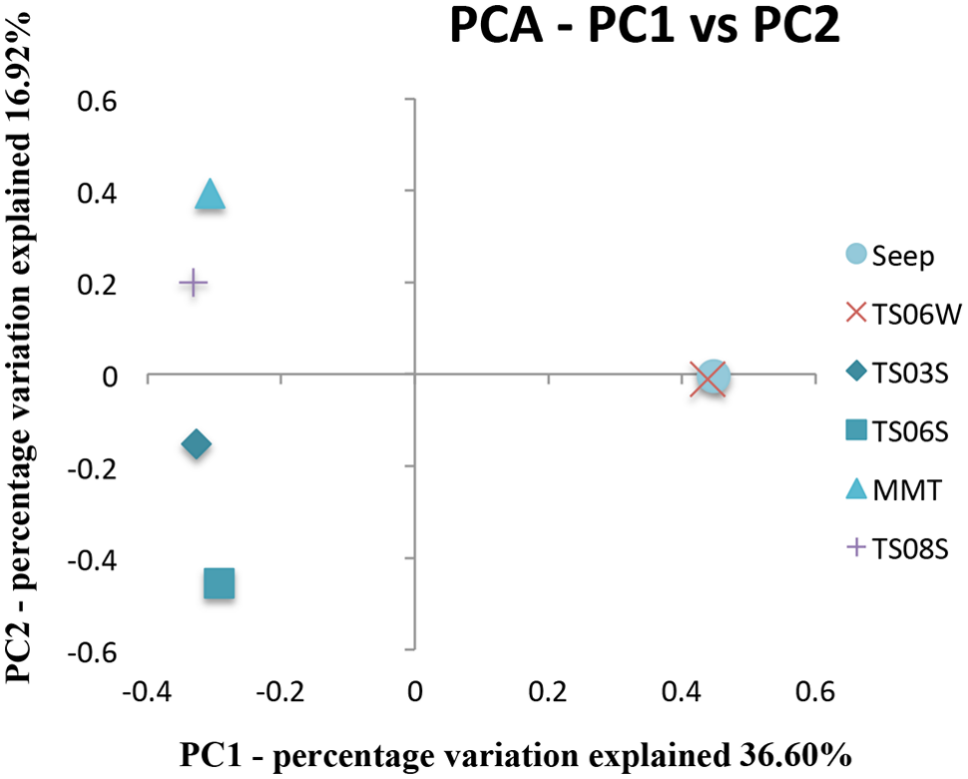
**Principal component analysis (PCA) and jackknife environment cluster analysis of all *amoA* genes from various microbial habitats in the Red Sea**.

Five types of habitats of archaeal ammonia oxidizers were examined herein (**Figure 5**). AOA communities were more similar within habitats than among habitats, as deduced from NMDS, UniFrac, Anosim, and Libshuff analyses (**Figures 5** and **6**; **Table 3**). For example, the clone libraries of the *amoA* gene from marine water shared more similarities with those from the cold seep and clustered together, as supported by UniFrac analyses (**Figure 6**). Based on the OTUs defined in each sample, NMDS plotted almost all of the clones from the seeps and marine water together. These samples were separated from those retrieved from marine sediments and the microbial mat (**Figure 5**). Although the water from the cold seeps had a higher salinity than normal marine water, similarities were observed among the communities of archaeal ammonia oxidizers between the cold seep and normal marine water (**Figures 5** and **6**).

**Table 3:** Anosim and Libshuff analyses to compare the similarity of archaeal ammonia oxidizer communities between two clone libraries based on *amoA* gene sequences.

Comparison	Anosim		Libshuff		
	
	*R*-value	*P*-value	dCXYScore	dCYXScore	Significance
TS03S-TS06S	–0.00430062	0.576	0.00068928	0.00083971	0.4453
TS03S-TS06W	0.772233	<0.001	0.10188576	0.10530356	<0.0001
TS03S-TS08S	–0.00203718	0.481	0.00100324	0.0050561	0.1691
TS03S-Top	0.0981375	<0.001	0.00100724	0.00607718	0.0006
TS03S-Seep4	0.75163	<0.001	0.08077245	0.0915377	<0.0001
TS06S-TS06W	0.749073	<0.001	0.09460696	0.11381637	<0.0001
TS06S-TS08S	–0.0531915	0.816	0.00172539	0.00138704	0.4792
TS06S-Top	0.103425	<0.001	0.00132125	0.00821533	0.0003
TS06S-Seep4	0.726427	<0.001	0.07498732	0.09949689	<0.0001
TS06W-TS08S	0.91786	<0.001	0.1110918	0.10863281	<0.0001
TS06W-Top	0.746849	<0.001	0.11072986	0.10837393	<0.0001
TS06W-Seep4	–0.0340392	0.792	0.00464578	0.01060209	0.0074
TS08S-Top	–0.0489684	0.824	0.00303009	0.00709402	0.055
TS08S-Seep4	0.931857	<0.001	0.08617571	0.09733566	<0.0001
Top-Seep4	0.692334	<0.001	0.08725311	0.09484944	<0.0001

## Discussion

An interaction between the fluid composition and microbes in the cold seep environment has been observed in some active cold seeps (Levin, 2005; Suess, 2010). The responses of microbes, such as microbial nitrogen utilizers, to the organic-rich fluids, however, has not been well investigated. In the present study, we confirmed the previously described presence of groups of archaeal ammonia oxidizers in a cold seep (Dang et al., 2010) based on the *amoA* gene and revealed clear changes in microbial communities along the environmental gradient from the seep vent to the cold seep brine pool and its surroundings (**Figures 2** and **4**).

Based on the 16S rRNA gene analysis, most of the reads from the microbial habitats could be sorted into known phyla. At the genus level, however, a variety of uncultured groups were assigned as endemic in different habitats in the Thuwal seeps, indicating a unique repertoire of novel microbial lineages. The potential ecological function of these groups is unclear, and further investigations using cultures or single cell genomics methods are necessary. For example, the OPB95 group affiliated with the candidate division OP8 was identified as the dominant group in marine sediment from the brine pool (Hugenholtz et al., 1998). MBGD and dhveg-1 (Thermoplasmata) were relatively abundant in microbial mats and in normal marine sediments in the present study. A previous study has already described the role of MBGD in protein remineralization in anoxic marine sediments (Lloyd et al., 2013). Altogether, these uncultured microbial groups support the need for additional functional studies.

The transition of archaeal ammonia oxidizers from the seep vent to the surrounding microbial mats, marine sediments, and water was clearly observed. Although relatively high concentrations of ammonia were recorded in the brine sediments, a high abundance of ammonia oxidizers was not observed. This result could be attributed to the low oxygen concentration; archaeal ammonia oxidizers still require oxygen as an electron acceptor. Another potential explanation could be a strong effect of salinity in determining AOA community structure patterns. Previous studies of prokaryotic phylogenies have revealed a clear separation between freshwater and marine lineages (Lozupone and Knight, 2007; Auguet et al., 2010; Cao et al., 2013), suggesting that salinity is one of the most important evolutionary barriers preventing frequent environmental transitions (Logares et al., 2009). In the present study, the various levels of salinity in different areas of the brine pools and cold seeps (Wang et al., 2014b) may have had a great influence on the evolution of AOA. In addition to the salinity, the concentrations of inorganic nitrogen-related compounds could represent another influential environmental parameter because the diversity indices changed along the concentration gradient of ammonia from the seep vent to the surrounding areas.

A lower abundance but a high diversity of unclassified Thaumarchaea was detected in the marine sediments and microbial mats, consistent with the findings of previous studies (Dang et al., 2010; Cao et al., 2011a, 2012). However, the marine water and seep vent were largely composed of uncultured Thaumarchaea with a low diversity but a high abundance, which is in agreement with the low diversity indices observed for the *amoA* sequences of marine plankton in a recent global-scale study (Cao et al., 2013). It is possible that habitats with low concentrations of substrate contain more diverse archaeal ammonia oxidizers, while habitats with high concentrations of substrate have a low diversity but a high abundance of this group. One interesting observation was that the *amoA* gene sequences from the normal marine sediments and the top of the microbial mat in the Red Sea shared a high similarity with sequences from the South China Sea but with limited sequences from other marine sediments (Cao et al., 2011a, 2012; Dang et al., 2013) (**Figure 4**). The ecological significance and allopatric distribution of this group of archaeal ammonia oxidizers in the marine sediments of Red Sea and South China Sea remains enigmatic and should be assessed in future studies.

In general, the advective supply of methane leads to dense microbial communities with high metabolic rates and anaerobic methane oxidation that is presumably coupled to SR and thus facilitates the formation of carbonates and generates extremely high concentrations of hydrogen sulfide in pore waters (Stokke et al., 2012; Maignien et al., 2013; Vigneron et al., 2013). Nonetheless, methane oxidizers were less abundant in the present study, which was in stark contrast to the typical cold seeps. However, fairly low concentrations of methane in the Thuwal seeping water (unpublished results) might explain the absence of microbes that oxidize methane in this cold seep ecosystem. The reduced abundance of methane oxidizers and scarcity of methane support the ubiquity of the cold seep in the Red Sea.

Overall, our analysis provides further insight regarding the changes in the microbial communities that reside in different habitats of the cold seep ecosystem in the Red Sea. We found (1) an unexpected number of unclassified microbial groups that contained several groups from candidate divisions with unknown ecological functions, (2) community structures that tended to be grouped by habitat types and salinity and oxygen concentrations appeared to be the driving force in shaping community structures, and (3) archaeal ammonia oxidizers in seeps and marine water differed from those in microbial mats and two types of marine sediments, consistent with many previous studies.

## Conflict of Interest Statement

The authors declare that the research was conducted in the absence of any commercial or financial relationships that could be construed as a potential conflict of interest.
