# Supplementary material for: Microbial community changes along the active seepage site of one cold seep in the Red Sea
Source: Front Microbiol. 2015 Jul 21;6:739. doi: 10.3389/fmicb.2015.00739 (PMC4523032; doi:10.3389/fmicb.2015.00739)

Supplementary material

Figure S1 Diversity indices (Shannon, Chao1, PD and OTUs) of 16S rRNA gene in all samples from the Red Sea.

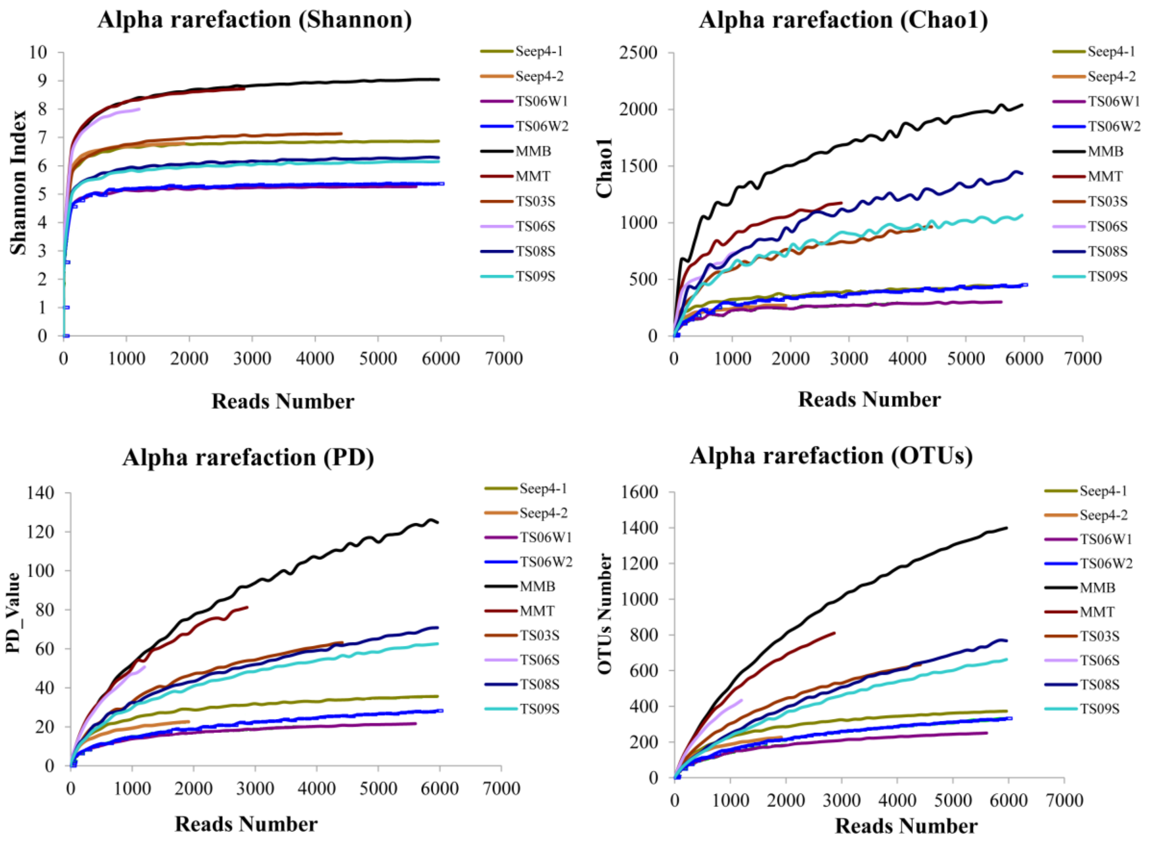

Figure S2 Taxonomic classifications of microbial reads on the phylum level retrieved from different habitats in the Red Sea on the basis of 16S rRNA gene pyrosequencing data.

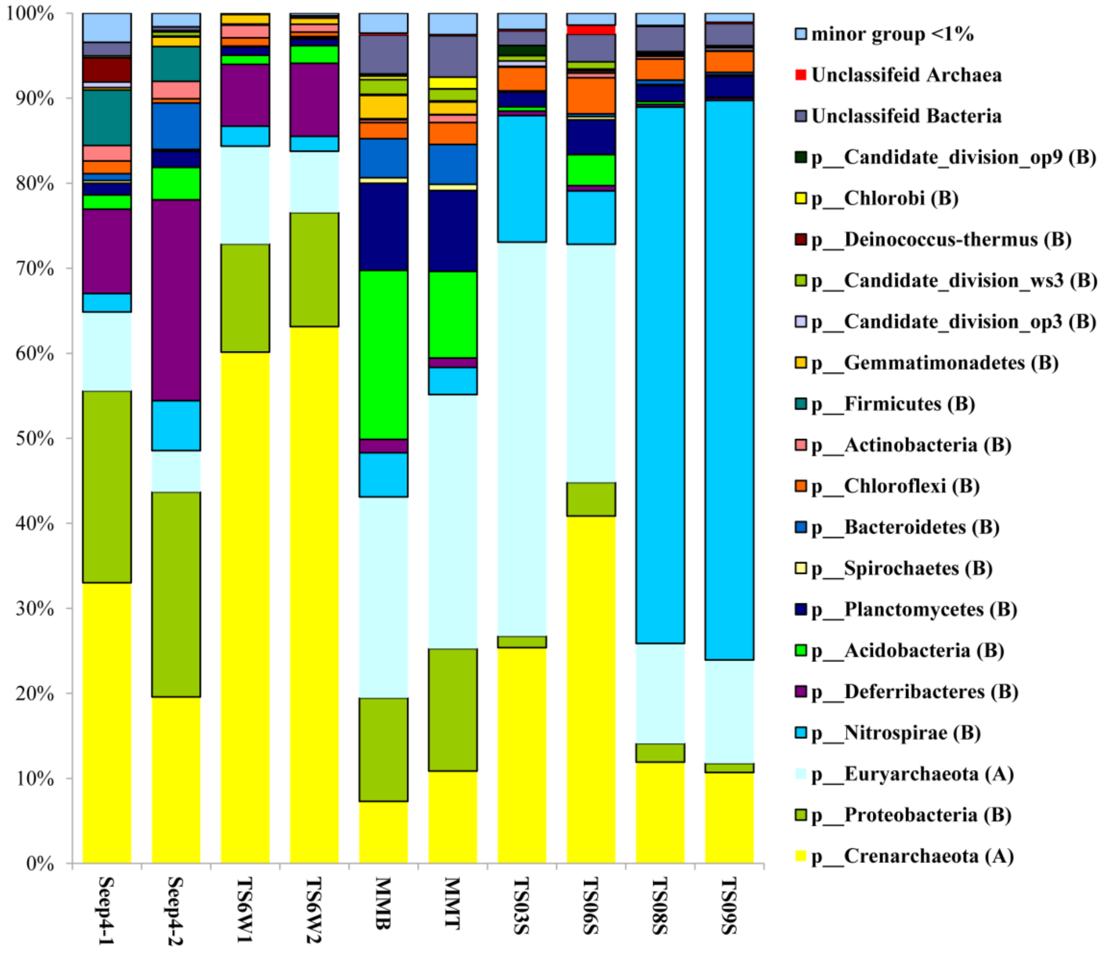

Supplement: Supplementary file 1 [file Image_1.PDF]
